# “Those are things for married people” exploring parents’/adults’ and adolescents’ perspectives on contraceptives in Narok and Homa Bay Counties, Kenya

**DOI:** 10.1186/s12978-021-01107-w

**Published:** 2021-02-23

**Authors:** Jefferson Mwaisaka, Yohannes Dibaba Wado, Ramatou Ouedraogo, Clement Oduor, Helen Habib, Joan Njagi, Martin W. Bangha

**Affiliations:** 1 College of Health Sciences, University of Ghana, Accra, Ghana; 2 African Population and Health Research Center-APHRC, Nairobi, Kenya; 3 Independent consultant, Nairobi, Kenya

**Keywords:** Adolescent girls, Contraception, Misconceptions, Parents, Perspectives, Kenya

## Abstract

**Background:**

Contraceptive use among adolescent girls is low in many sub-Saharan African countries including Kenya. Attitude and perspectives about contraception of community members including adolescent girls themselves may be likely to limit contraceptive use among adolescent girls. This study was conducted to explore and compare adults’/parents’ and adolescent girls’ narratives and perspectives about contraception in Narok and Homa Bay counties, Kenya.

**Methods:**

Qualitative data from 45 in-depth-interviews conducted with purposively selected consenting adolescent girls aged 15–19 was used. Additionally, twelve focus group discussions were held with 86 consenting adults conveniently recruited from the two counties. All discussions were conducted in the local language and audio recorded following consent of the study participants. Female moderators were engaged throughout the study making it appropriate for the study to solicit feedback from the targeted respondents.

**Results:**

Findings highlighted adults’ perceptions on adolescents’ sexuality and the presence of stringent conceptions about the side-effects of contraception in the study communities. Some participants underscored the need for open contraceptive talk between parents and their adolescent girls. Four main themes emerged from the discussions; (i) Perceptions about adolescents’ sexuality and risk prevention, (ii) Conceptions about contraception among nulligravida adolescents: fear of infertility, malformation and sexual libertinism, (iii) Post-pregnancy contraceptive considerations and (iv) Thinking differently: divergent views regarding contraceptives and parent/adolescent discussion.

**Conclusions:**

Our findings suggest the need for increased attention towards adolescents and their caregivers particularly in demystifying contraceptive misconceptions. Programmatic responses and models which include the provision of comprehensive sexuality education and increased access to and utilization of SRH information, products and services through a well-informed approach need to be well executed. Programmatic efforts like SRH community education should further seek to enhance the capacity of parents to discuss sexuality with their adolescents.

## Plain English summary

Contraceptive use provides multiple benefits including preventing unintended pregnancies and reducing abortion incidences. Despite these known benefits, adolescent girls’ contraceptive use is the lowest in Kenya compared to other sexually active age-groups. Studies have documented several factors associated with this low use of contraceptives among sexually active adolescent girls including; adolescent girls’ poor contraceptive knowledge, myths and misconceptions associated with modern contraceptives and judgmental tendencies of adults towards adolescents’ sexual and reproductive health (SRH) services particularly on access to modern family planning methods. Although significant progress has been made in understanding adolescent girls’ contraception behaviour and factors contributing to their low uptake, few studies if any have compared both parents’, other adults in the community and adolescent girls’ perspectives regarding contraception. We therefore sought to explore and compare adolescent girls’, their parents’ and other adults’ narratives and perspectives about contraception in Kenya. Using data from in-depth interviews conducted with 45 adolescent girls aged 15–19 and 12 focus group discussions held with 86 adults, we established that contraceptive behaviors among adolescent girls entail a balance between adults’ perceptions towards adolescents’ sexuality and perceived effectiveness of modern contraceptives. This suggests that there is a need for increased sexual and contraceptive talk between parents/caregivers and their adolescent girls particularly in demystifying contraceptive misconceptions and to help parents understand their daughters’ sexual behaviors.

## Background

Globally, approximately 16 million girls aged 15–19 years and two million girls below the age of 15 become pregnant every year, with the poorest regions of the world estimated to have four times higher prevalence of adolescent pregnancies compared to their counterparts in the higher income regions [1]. Estimates from the 2014 Kenya Demographic and Health Survey (KDHS), suggest that 18% of girls aged 15–19 have already had a birth or are pregnant with their first child [2]. Unmet need for family planning and early sexual debut have been associated with teenage pregnancies, especially from the poorest households [3–5]. Whilst the 2014 KDHS established that the median age at first sexual intercourse among women in Kenya was 18 years [2], county-level variations exist with counties like Migori, Homa Bay and Samburu having a median of 15.5, 15.7 and 15.7 years respectively [2]. Other variations in sexual debut have been observed among adolescent girls and young women with regards to place of residence, educational levels and income inequalities [2, 6–8]. The 2014 KDHS reported a contraceptive prevalence rate (CPR) of 40.2% (any method) and 36.8% (any modern method) among married adolescents aged 15–19. CPR among sexually active unmarried adolescents is estimated at 49.3% for modern contraceptives and 50.1% for any method [2]. Although contraceptive use among sexually active unmarried adolescents is slightly higher, they still have a high unmet need for contraception. Inevitably, the resultant effect of early sexual debut coupled with low contraceptive prevalence rates is poor SRH outcomes, including early child births; most of which are either mistimed or unintended [7–9]. Moreover, adolescent girls with unintended pregnancies may opt for unsafe abortions further complicating their SRH outcomes. For instance, it is estimated that 41% of annual unsafe abortions in developing countries occur among women aged 15–25 years with 70% of complications due to unsafe abortion occurring among girls below 20 years of age [10]. A nationally, representative cross-sectional study on abortion complication severity and associated factors in Kenya established a high abortion complication fatality rate; adolescents aged between 12 and 19 years constituted approximately 34% of patients presenting for post-abortion care in Kenya with almost half, 47% being unmarried [11].

Contraceptives offer multiple direct and indirect benefits; including preventing unintended pregnancies, reducing abortion incidence and lowering the burden of mortality and morbidity associated with pregnancy complications and child births especially among adolescent girls [12]. Despite these broad benefits, adolescent’s contraceptive use is lowest compared to other sexually active age groups [1]. For Kenya, deterrents of adolescent uptake include the adolescents’ own lack of awareness, misperceptions and issues of confidentiality as well as parental consent [13–15]. In terms of provider-related issues, attitudes, misconceptions and limited training are stringent factors [9, 16]. Notably, parental disapproval mostly due to exaggerated and misperceived side effects propagated by their societal inclinations have been evinced to contribute to the low uptake of contraceptives among adolescent girls [14, 17]. However, knowledgeable parent-adolescent interactions can foster related thoughts and considerations associated with the effectiveness of modern contraception which may ultimately minimize inaccurate beliefs about modern contraception. Studies have shown that mother-daughter SRH discussions increase safer sexual behaviour among adolescents, with a likelihood of increasing positive attitudes towards contraception [17–20]. Parents can therefore provide effective platforms for SRH discussions which their adolescent daughters may emulate in their own sexual relationships [19]. The urgent need to encourage family and community discussions that dispel myths about contraceptive use cannot be overemphasized. Understanding parental and adolescents’ contraceptive concerns and involving parents in adolescent pregnancy prevention programmes is vital as studies have shown that low parental communication and support is a key predictor of teenage pregnancies in sub-Saharan Africa [21]. Whilst some parents have claimed that they have been engaged in sexuality education of their adolescents, in reality, such discussions are often barely communicated tacitly through intimation. This is because parents usually fallaciously believe their teenagers are not sexually active [22] and do not want to ‘arouse’ their sexual feelings through explicit SRH education involving contraceptive talk. Realistically, most adolescents are already forming ideas and engaging in sexual practices during this period [3, 10].

Few studies globally and in particular sub-Saharan Africa, have compared both parents’ and adolescent girls’ narratives with regards to contraception [17]. There is evidently a need to explore and understand how parents and their adolescent daughters acquire and perceive contraceptive knowledge, attitudes and behaviors. This includes parental perceptions and receptivity of contraceptive discussions in order to effectively demystify contraceptive misperceptions and increase adolescents’ CPR. This qualitative study sought to explore and compare adults’/parents’ and adolescent girls’ narratives and perspectives about contraception in Narok and Homa Bay counties, Kenya.

## Methods

### Study setting

This study was part of a larger evaluation which aimed at providing baseline information on the key aspects of a digital adolescent SRH intervention, ‘In Their Hands’ (ITH). ITH in Kenya was a digital health program that aimed to increase adolescents’ use of high-quality SRH services through targeted interventions. The ITH programme provided SRH information while promoting adolescents’ use of contraception, pregnancy tests and testing for sexually transmitted infections (STIs) including HIV. The project was implemented in eighteen counties in Kenya, prioritized based on their burden of teenage pregnancies, unmet need for contraception among adolescent girls and incidence of STI and HIV infections. For the evaluation component, two counties were selected from the counties where the intervention had not begun at the time of the baseline study, one from Nyanza region (Homa Bay) and another from Rift Valley (Narok).

### Study design

This was a qualitative study involving adolescent girls, parents/adult caregivers and community health volunteers (CHVs). In Kenya, CHVs are lay members of the community sharing ethnicity, language and life experiences of the communities they serve. One needs to have a minimum of primary level education to qualify to be a CHV. They are given basic health training to support their community to improve their general health status including maternal health, nutrition, basic hygiene and other behavioral health interventions. The full training curriculum takes approximately three months. We conducted 45 in-depth-interviews (IDIs), 20 in Homa Bay County and 25 in Narok County with purposively selected adolescent girls aged 15–19 who were usual residents (lived in the study communities at least six months preceding the study). Additionally; eight focus group discussions (FGDs), four per county with parents/adult caregivers (all mothers); and another four (two in each county) were held with CHVs affiliated to health facilities that were selected for the ITH programme. Eligibility criteria for other adult FGD participants included having an adolescent girl aged 15–19 years. Participants’ characteristics varied by age, level of education, occupation, marital status and parity. Discussions were conducted in the local language and audio recorded following consent of the study participants. Interviewers were trained to facilitate the discussions and were provided with semi-structured interview and discussion guides for the IDIs and FGDs respectively. Face to face interviews were held with adolescent girls, and in groups for mothers and CHVs. IDIs were used to explore adolescent girls’ SRH concerns and services seeking behaviors including their views on contraception. The FGDs with the community (caregivers and parents) and CHVs were used to explore the community’s attitudes towards adolescent sexuality and their concerns on SRH services for adolescents including contraception for adolescent girls. To minimize discomfort and any unforeseen embarrassment surrounding the study topic, female moderators were engaged throughout the study. Additionally, female moderators freely and easily unlocked the real issues associated with adolescent girls’ SRH concerns thereby facilitating free and open feedback from the targeted respondents.

### Data management and analysis

Audio recordings from the IDIs were anonymized, labelled with unique identifiers and deleted from digital recorders once transcription was completed. The discussions were transcribed verbatim, translated into English, coded and analyzed thematically using NVivo version 12. A “thematic analysis” approach was used to organize and analyze the data, and to assist in the development of a codebook and coding scheme. A preliminary code book was developed using the interview guide and a set of IDIs and FGDs transcripts, and discussed among the research team. Data was analyzed by first reading the full transcripts of FGDs and IDIs, familiarizing with the data and noting the emergent themes and concepts. A thematic framework was developed from the identified themes and sub-themes, and then used to create codes for the raw data. Our qualitative analysis followed a pattern of association on the key identified themes, particularly focusing on narratives related to adolescent girls’ contraceptive use. “Misconceptions about contraception” in this study are defined as “widespread views about the effects and purpose of contraceptives that are not supported by any scientific evidence” [23], “sexual libertinism” on the other hand refers to the practice of adolescent girls pursuing their own personal sexual desires while disregarding societal expectations and norms. Our analysis and findings are presented in accordance with the Standards for Reporting Qualitative Research guidelines (SRQR) [24].

### Ethical considerations

The protocol for this study was reviewed by African Population and Health Research Centre’s scientific and ethics committee and adjudged to be scientifically sound. The institutional review board (IRB) approval for the study was given by the AMREF Health Ethics and Scientific Review Committee (AMREF-ESRC P499/2018). Research permit for the study was granted by Kenya’s National Commission for Science, Technology and Innovation (NACOSTI). Additional approval was obtained from county and sub-county commissioners, Ministries of Health and Education in the respective counties; and other local administrators including, Chiefs, Assistant Chiefs and Village Elders. All participants gave written informed consent to participate in the study. For adolescents aged below 18 years and not emancipated, both parental/guardian consent and adolescent assent were obtained before starting the interviews.

## Results

The socio-demographic characteristics of the 45 adolescents are presented in Table 1. Most of the adolescent girls interviewed were below 18 years (64% in Narok and 65% in Homa Bay). Majority of our respondents were single at the time of the study with one in five in Homa Bay County (20%) and one in ten in Narok Country as married respondents.

**Table 1:** Sociodemographic characteristics of adolescent girls by county

Variable	Narok	Homa Bay
*Age group*		
Under 18 years	16	13
18–19	9	7
*Highest level of school attended*		
Primary	14	9
Secondary and above	5	8
Missing*	0	3
*Marital status*		
Married	3	4
Single/never married	22	16
*Parity*		
Has a child/currently pregnant	9	10
No child	16	10
*Activity status*		
In school	11	11
Unemployed	14	9
Total	25	20

We also conducted a total of 12 focus group discussions with 33 CHVs and 53 parents and caregivers of adolescents in both Homa Bay and Narok counties. The characteristics of the FGD participants are presented in Table 2. The majority of the CHVs and parents had a primary level of education. Occupation wise, most of the CHVs were involved in voluntary work as community health workers while the majority of the parents were involved in farming and business.

**Table 2:** Sociodemographic characteristics of adult respondents (CHVs and parent/caregivers)

Characteristics	CHVs	Parents/caregivers
*County*		
Homa Bay	10	26
Narok	23	27
*Highest level of school attended*		
No education	2	19
Primary	19	27
Secondary and above	12	7
*Occupation*		
Business	9	20
Farming	8	32
Volunteer work—Community Health	13	0
Others (tailoring, pastor, housewife)	3	1
*Residence*		
Urban	18	22
Rural	15	31
Total	33	53

The analysis of interviews with adolescent girls, parents/adult caregivers and CHVs revealed their perceptions on adolescents’ sexuality and alternatives to protect them from sex related consequences and perceptions on contraceptive use through four main themes; (i) Perceptions about adolescents’ sexuality and risk prevention, (ii) Conceptions about contraception among nulligravida adolescents: fear of infertility, malformation and sexual libertinism, (iii) Post-pregnancy contraceptive considerations and (iv) Thinking differently: divergent views regarding contraceptives and parent/adolescent discussion.

### Perceptions about adolescents’ sexuality and risk prevention

Both parents, CHVs, and adolescents described early sexual debut as “dangerous’’ “bad’’ ‘’wrong’’, with disastrous repercussions such as: diseases (STIs), HIV, pregnancy and dropping out of school and eventually getting married as a second wife, among other negative effects. This is explained by adolescents and parents in the following quotes:

Parents tell you that such things are bad when you are in school, you can be pregnant, you can be sick and you can have psychological problems which can lead to your failure in school (15-year-old, student, single, no child, rural Homa bay).

We tell them; sex is not for children. We tell them that there is a time for it, and this is not the time to have sex. (Parents FGD participant, rural Narok).

As a result of these perceptions about early sex and romantic relationships, participants reported low parental engagement, evidenced by the lack of, or inadequate parent-adolescent discussions. Indeed, most participants pointed out the lack of discussions between parents and adolescent girls. Adolescents indicated facing difficulties in talking to their parents as they tend to be harsh.

If the parent is harsh, you can be afraid of asking her some things. (17-year old adolescent, single, with one child, Narok).

Parents expressed discomfort talking about sex with their children because of the taboo ascribed to such discussions, and feeling incompetent to handle such topics. As such, parents did not talk about these issues until there was perceived danger. Most discussions on sex with adolescent girls were therefore necessitated by perceived danger such as signs of interest from or toward the opposite sex, associating with older adolescents or those considered to be 'bad company' or fear of pregnancy following physical markers of pubertal development.

The girl at this period [adolescent age] becomes very vulnerable as she can become pregnant at any time, so we advise them that when she experiences monthly periods for the very first time she should be very careful with men and boys because she is at risk of getting pregnant, that is what we usually tell them (Parents’ FGD Participant, Narok East).

I don’t know whether it is a loophole or a weakness on the parents because most parents wait till their daughters engage in sex before we can talk about it. So mostly you find that we talk to our girls after the milk is spilled… so we only talk after a child has messed and that’s a weakness we have as parents (Parents’ FGD Participant, Ndhiwa town).

Discussions on sex were gendered, with mothers discussing with their daughters and sometimes sons and fathers not engaging in such conversation.

For me I am a mother to boys and boys are different from girls, so I usually advice boys on education issues to work hard in school, for girls I advise them to take care of themselves and protect themselves from sex and pregnancy (Parents’ FGD Participant, Narok).

During the FGDs, parents mentioned that sometimes they relegate the responsibility of discussing sex to other adults in the community, most often to CHVs, due to their lack of knowledge and skills of communicating with adolescents.

We have a CHV here, she comes and counsels our young girls and the new mothers. When they come, as a parent, I have to respond and give her the chance to talk to the child, but if she also wants to consult me, I may also wait and listen, why – because as a CHV, there are things they know about counseling which they may know more than I do as a parent (Parents’ FGD participant, Kasipul West).

Moreover, the discussions tend to have a strong focus on abstinence until marriage or until completing school, as the most effective way to avoid the dangers associated with sex. However, participants, especially adults, acknowledge the fact that current school-going adolescents are more sexually active than ever, and vulnerable to both early unintended pregnancies and STIs. In this context, condoms appeared to have a wider social acceptability by both adolescents and adult participants for preventing premarital pregnancies as well as sexually transmitted diseases including HIV. For instance, one adolescent in Narok was advised by her aunt to use a condom if she happens to have sex to prevent pregnancy:

She told me that when you do it [have sex] with a boy, you should use a condom so that you don’t become pregnant – that’s what she told me and my sister (15-year old adolescent, single, with no child, Narok).

Another rationale underlying this preference for condom use is the prevailing conceptions associated with the use of hormonal contraceptives methods by adolescent girls as discussed in the next section.

### Conceptions about modern contraception among nulligravida adolescents

Findings from this study established that contraceptive behaviors among adolescent girls entail balancing between the perceived effectiveness of modern contraceptive for preventing pregnancies with perceptions associated with particular methods. Moreover, adolescent girls’ preference for contraceptives appear to be motivated by perceived health effects associated with hormonal methods and sexually transmitted infections (STIs) including HIV. Modern contraceptive methods appeared to be the most effective way to prevent unintended pregnancies among sexually active adolescent girls. However, according to participants, hormonal contraceptives introduce substances in the body mechanisms which may have some consequences such as infertility or fetal malformation. Indeed, there was a seeming consensus among participants, regardless of their area of residence, that the use of contraceptives among adolescent girls, especially unmarried and nulligravida, may interfere with their future fertility and cause inability to conceive later in life. Adolescent girls reported having heard the same from various sources, including from parents as clearly captured in a conversation with one adolescent girl below;

Interviewer: Have you ever discussed contraceptives with her?

Respondent: On that, she warned me never to use.

Interviewer: Why did she tell you so?

Respondent: She [mother] told me they lead to some problems. You may fail to get pregnant and there are also other diseases that they may bring to the body. (17-year old adolescent, student, single, no child, rural Homa Bay).

One pregnant adolescent portrayed mixed thoughts as to whether she would use contraceptives in future or opt for abstinence. She first indicated her intentions to use contraceptives in future to avoid other unplanned pregnancies.

Interviewer: So, when you get your child, is there any way that you will prevent yourself from getting pregnant again?

Respondent: Yes.

Interviewer: What will you do?

Respondent:I will go for family planning. (15-year-old adolescent, five months pregnant during the study, rural Narok).

However, the girl immediately retracted her decision to use contraceptives due to perceived infertility fears and indicated that she would instead opt to abstain and maintained that position to the end of the interview.

Respondent: I will not have sex. When you hear what people are saying you cannot use them [contraceptives]

Interviewer: What did you hear?

Respondent: For example, you can never give birth again (16-year old adolescent, single, out of school, with a child, rural Narok).

This is a strong and widespread position to the extent that it creates fear among adolescent girls, given the crucial role of motherhood in women's lives. According to the participants, contraceptive use is meant to prevent early unintended pregnancies in order to give them time to be ready for motherhood once they complete school, get married, and/or get employed. However, the fear of using modern contraceptives (to prevent pregnancy) is perceived to cause infertility thereby impeding their future motherhood status appears stronger than having a premarital birth. Compared to contraceptive use, early pregnancies seem to be the preferred alternative. Adult caregivers opined that adolescent girls’ use of contraceptives will affect their fertility in the future, supported with anecdotes of other girls’ difficult encounters with childbirth after marriage;

We have seen many girls who have used family planning methods and at the time they got married they were unable to give birth due to the drugs they have been using. These drugs damage their wombs. We will mislead our daughters if we advise them to use family planning, it is even better for a girl to give birth while young (laughing) than to get such effects from those drugs (Parents’ FGD participant, rural Narok).

Surprisingly, this negative perception about contraceptives was also common among the CHVs interviewed. CHVs are supposedly knowledgeable people trained to educate communities on various health related topics and create the link with the health system in Kenya. However, they seem to share the same sentiments against modern contraception while some instead advanced their preference for condoms. Asked why some CHVs focus on condoms during their SRH education for adolescents, one CHV stated:

You know when these children who are 15 years [old] use family planning methods, they affect them in a negative way, like if you have not given birth one may end up getting no child because of family planning methods so we advise them to use condoms (CHVs’ FGD participant, rural Narok).

This stance was so deeply-seated that one CHV also confirmed the view equally voiced during the FGDs with parents to the effect that they would rather have their daughters give birth than have them on contraceptives. A typical comment included:

It is even better for your daughter to give birth and take care of her baby rather using contraceptive methods. (CHVs FGD participant, urban Narok).

It is however important to note that even though CHVs were more comfortable providing information on condom use, compared to providing information on hormonal contraceptives, such information was mostly provided to male youth. Moreover, even though CHVs demonstrated more acceptance towards condom use, there were elements of misinformation with an intention to discourage young people from engaging in sex. For instance, one CHV in rural Narok indicated, "we tell them that they can use condoms, and we also tell them that condoms are not 100% effective". As such, even condom information was still geared towards promoting abstinence in an indirect manner, particularly for younger adolescents.

Yes, we usually tell those young teenagers how they can protect themselves…first we usually tell them to use condoms when they meet their friends. So we usually tell them not to move with those guys without condoms because they can really get pregnant, those diseases like gonorrhea, they were there. So we usually tell them to protect themselves. (CHVs FGD participant, rural Homa Bay).

In addition to the issue of infertility, participants felt that contraceptives are meant for married women to either delay a pregnancy (giving time to her baby to grow) or stop having children when the desired number is reached. In line with that, one adolescent girl mentioned that parents were not comfortable discussing contraception with them; and concerns about contraceptives were tacitly highlighted by their parents who opined that contraceptives were only suitable for married women.

No- parents do not want [to discuss] issues of family planning because right now I am still a student. Those are things for married people. (18-year-old, student, single, no child rural Homa Bay).

Fetal malformations as a result of contraceptive use was also alluded to by adolescents implying fear that infants born to girls using contraceptives might experience birth defects. Potential malformation as a result of injectables for instance was mentioned by one of the adolescents in Narok:

If you use injections [contraceptives] you can get a child without arms, and legs, I heard that in school (19 years old, form 4 leaver, single, no child, urban Narok).

Perceptions about contraceptive use and sexual libertinism were also reported by participants, mainly parents and CHVs. Several parents and CHVs mentioned that contraceptive use by adolescents would lead to an increase in risky sexual behaviors among adolescent girls. According to the participants, talking adolescents into using contraceptives will encourage them to engage in sex freely since the fear of pregnancy is eliminated, therefore providing them an open ticket to engage in sexual activities. These sentiments appeared widespread among a majority of the adult respondents in both rural and urban areas.

About family planning, those adolescents of that age between 15 to 19 years, we never talk to them to use family planning methods. We only teach women about family planning because when we tell adolescents about family planning methods it will be like we are encouraging them to engage in sex because they know they will not get pregnant. So it will be like we are giving them the freedom of engaging in sex. (CHVs’ FGD Participant, Rural Narok).

For us, we don't tell them to use family planning methods. We really discourage that because they are still children and you will never get a Maasai telling his/her daughter to go for family planning to prevent pregnancy, it is even better for her to give birth and the mother will take care. So it is hard for parents to tell their children to go for family planning unless the girl just goes for it without their parents' knowledge. (Parents’ FGD Participant, urban Narok).

In addition to girls being promiscuous, the concerns were also about the lack of protection against infections and diseases. One adolescent girl reported that her mother’s judgmental attitude was maintained by her belief that contraceptives would create a false sense of sexual safety, that may in turn encourage risky sexual behaviors and expose the girl to sexually transmitted infections (STIs).

she [my mother] only tells me to abstain to avoid all those, ‘I cannot take you for family planning because after I take you, you will know that you are protected from pregnancy but you won’t be safe, so you will be sure you cannot get pregnant and so you will not fear the diseases as well’. So they tell me to abstain. (15-year-old, student, single, no child, rural Homa bay).

Similarly, one parent in Narok county shared the same views that contraceptives would encourage risky sexual behaviors while they lack dual protection from both pregnancies and STIs.

If you tell the girl about family planning, you will be encouraging her to associate with boys freely. It means that I have told her the ways of not getting pregnant. But you have not prevented her from being infected. So that is not the best way because it will not prevent infection, the best way is to teach them to be God fearing so that when they grow up they will be free to choose. (Parents FGD, Urban Narok).

### Post-pregnancy contraceptive considerations

Despite reluctance to contraceptive discussions with their nulligravida adolescent girls, both parents and CHVs were willing to discuss contraception after an established pregnancy and/or childbirth. Pregnancy facilitated an acknowledgment that the girl was sexually active and could get pregnant again, hence the need to take preventive action particularly to help them complete school without getting pregnant again. Moreover, with the adolescent girl becoming a mother, this ‘confirmed fertility’ and the fears of infertility due to contraceptive use were alleviated.

Yes, it is true they will get another one [pregnancy] if you are not careful. For example, there was one who got pregnant when she was in form 2 and her mother decided to give her an injection for family planning. You know at least she has given birth so it might not be a big deal, those that had already gotten children are better if injected as compared to those who have not. (Parents FGD participant, rural Narok).

For those who have already given birth and they are in school we tell them to use protection, family planning or condoms (CHVs FGD participant, rural Narok).

Allowing the adolescents to use contraceptives or introducing the contraceptive use becomes an alternative to the failure of an initial fear strategy; keeping silent about sex and avoiding all topics that may directly or indirectly teach the adolescent about sex. This introduction can also be explained by the fear on the part of parents of getting confronted with the burden of teenage pregnancies and related school dropouts by the adolescent girls, as explained by one of the parents below:

You know why you would – maybe a girl is in secondary and she can even give birth three times, so you also feel the burden because after a short period of time, she gets pregnant, she then stops using the fees as she is breastfeeding the child. Later you take her back to school … then gets pregnant again, so that is why you would tell her to look for family planning. (Parents FGD participant, rural Narok).

Additionally, where adolescent girls have had at least one child outside marriage, parents felt that it becomes necessary to introduce such girls to contraceptive use to help avoid the burden of having to take care of children born out of premarital pregnancies. This burden is faced both by the adolescent as a single mother to many children, and by her own parents who are taking care of their own children as well. One parent said the following about this:

So sometimes you will be forced that when a child has surpassed one, you will have to introduce – you have to tell her that my sister or daughter, if this is what it is, I cannot do anything more, let me introduce you to family planning and she gets an injection or an implant – any that she wants to help her prevent pregnancies because she is now a parent – a single parent. so she prevents pregnancies but go to school so that if she can finish her school – when you have two children, they need food, you also have your children as the parent – that is now a burden. So sometimes you just have to talk about family planning. (Parents FGDs participant, Urban Homa Bay).

These post-pregnancy contraceptive talks were confirmed by adolescents who even reported to be receiving pregnancy prevention information from other sources including their relatives and neighbors as explained by one adolescent girl in Homa Bay.

We had a neighbor who was on family planning and she told me to go for family planning so that I won’t have another child soon (19-year old, out of school due to pregnancy, with a child, Urban Homa Bay)

Similarly, other adolescent girls like this 18-year old girl from Narok got pregnant before they engaged with anyone on contraceptives related discussion:

We had not talked about it before I became pregnant but after I conceived, that’s when she [my sister] started talking to me about contraceptives so that when I give birth to this one, I don’t conceive again. (18-year-old, out of school due to pregnancy, single, urban Narok).

However, for her, the notion of post-pregnancy contraceptive discussions proved belated as she *“wish I was given this information earlier*” to avoid getting pregnant and dropping out of school.

### Thinking differently: divergent views regarding contraceptives and parent/adolescent discussion

Some parents/adult caregivers, particularly CHVs expressed the need to move beyond abstinence only messaging, to include information on different ways of experiencing sexuality, delaying sexual debut as well as the need for discussions on contraceptives with adolescents. Adult participants and adolescent girls alike were open to the idea that openness in SRH talk including contraception was essential and that time was ripe for such discussions to be embraced to reduce teenage pregnancies and its consequences such as school dropout.

Instead of solely focusing on abstinence children can learn different ways of expressing love, they can be taught to delay sex (CHVs FGD participant, urban Narok).

So what I tell them [is], if it is a must that they [boys] must have [have sex] with you, then you should use contraceptives – you should go to the facility for contraceptives – the types you like, that is available there for you. (CHVs FGD participant, rural Homa Bay).

Yes, we talk to them because sex…you don’t know the time they go to meet, so you just tell them to protect their life. But they will not tell you the time they will go to meet and have sex. Also most of them are school going so you only get few minutes to talk to them about contraceptives; when they come back from school and when they are in school some will take that advice seriously while others will not, but we usually talk to them about engaging in sex. (Parents FGD participant, rural Narok).

In most cases, the CHVs who expressed divergent and more accepting views of contraceptive use by adolescents, safe abortion and practicing sexuality in a safe and healthy manner had undergone training and value clarification. This was particularly evident during discussions with CHVs in urban Narok, as some had participated in training by different Non-Governmental Organizations including Marie Stopes Kenya. Some CHVs in urban Homabay who were involved in the (Determined, Resilient, Empowered, AIDS-Free, Mentored and Safe) DREAMS Programme, which promotes contraceptive use among sexually active adolescents also expressed more divergent and accepting views of contraceptive use by adolescents. This demonstrates the importance of training and value clarification in addressing provider attitudes towards adolescent sexual health. The discussion in that case will be both unveiling the subject of contraception and providing the adolescents with enough information to decide on the suitable methods depending on their situations and life goals (short term versus long term methods).

Some parents opined the need to adopt a top-down approach by first changing the mindsets of parents regarding contraceptives. This in one parent’s opinion will build the skills of parents and create harmony leading to positive SRH mindsets among both parents and their children.

In my opinion, let’s start from the top going down; that is starting from changing the parents themselves then going down to children, because when you start from changing the children going up, there will be a conflict of discussion. So it will be good [the project] to have one on one conversation with the parents first then meet the children after talking with the parents. When the girls see the changes in their parents, they will also change when they are advised. (Parents FGD, urban Narok).

Another parent alluded to the same view as she believed adolescent girls might not listen to parental advice owing to the perception by the adolescent girls that they are more educated compared to their parents.

Maybe she feels that she has gone to school but you never went so she doesn’t view you as one who went to school and can teach her. (Parents FGD, rural Homa Bay).

Importantly, when asked about if they talk to their adolescents about romantic relationships, some parent participants expressed their difficulties in initiating sexual talks with their adolescent girls. When probed further in an attempt to understand what exactly was difficult, it transpired that they lacked conversation starters for such discussions because of the lack of skill as well as the ‘taboo nature’ of sexual discussions leading to discomfort in talking to adolescent girls on the topic.

Respondent: It is hard [to discuss sex and relationships with adolescent girls].

Moderator: Why is it hard?

Respondent: issues about love between girls and boys – how will you start?

Moderator: So you find it hard to start? Why do you find it hard to start? That’s what I would like to know from everyone here?

Respondent: Currently if you want to ask the modern children, they start a quarrel with you. So you will just leave them but teach them so if they want to appreciate what you have told them then they will. But if you sit them down to teach them, sometimes they even dodge you, they won’t even listen to you. (Parents FGD, rural Homa Bay).

However, contrary to parents’ assertions regarding adolescent girls’ perceived ‘know it all’ attitude, one adolescent girl expressed concerns about unapproachable parents who may make it difficult for adolescents to approach them for advice on SRH issues.

*If the parent is harsh, you can be afraid of asking her some things.* (17-year-old, dropped out of primary school due to pregnancy, single, with one child, rural Narok).

Parents indicated a gap in forums and opportunities to enhance their parenting skills and recommended the need for initiatives through existing community spaces such as churches.

The church organizes seminars to teach us how to take care of our husbands, but not on how to take care of our children (Parents FGD, urban Narok).

## Discussions

This study explored and sought to contribute to a richer understanding of the dynamics between adolescents, their parents and community health volunteers with relation to contraceptive uptake among adolescent girls. The study shows that parents and CHVs in the two communities oppose early and pre-marital sex due to the perceived repercussions it causes on adolescent health and well-being such as: STIs including HIV, pregnancy and dropping out of school. As a result, there is a low parental engagement, evidenced by the lack of, or inadequate parent-adolescent discussions. It appears from the viewpoints of all participants in this study that stringent misconceptions about the side-effects of contraception are pervasive in the study communities and contraceptive behaviors among adolescent girls entail balancing between the perceived effectiveness of modern contraceptive for preventing pregnancies with misconceptions associated with particular methods. There is a general perception that hormonal contraceptives introduce substances in the body mechanisms which may have some consequences such as infertility or fetal malformation. Such misconceptions are widespread in Africa and are among the major barriers to modern contraceptive use [12, 16]. There is a seeming consensus among participants that the use of contraceptives among adolescent girls, especially unmarried and nulligravida, may interfere with their future fertility and cause inability to conceive later in life.

This finding is reflective of studies conducted in sub-Saharan Africa and other parts of the world which investigated the predictors of pregnancy among young people: lack of SRH knowledge, parenting and family related attitudes as well as low parental communication and support were key predictors of unmet needs for contraception [21, 25–27]. A study from Uganda investigating healthcare perceptions of family planning among adolescents established that lack of community SRH knowledge, myths and misconceptions among adolescent girls and health care providers’ attitudes were associated with non-use of contraceptives among adolescent girls [25]. Additionally, other studies outside Africa, specifically in Israel and the USA have also discovered high rates of misconceptions about contraception not only among adolescents and their caregivers but also among physicians [26, 27].

The issue of misconceptions about contraceptive methods appears ubiquitous worldwide. This occurrence presents a major barrier to the uptake of contraceptives as ostensibly, health workers, and in the context of this study, community health volunteers providing level one services and supporting community initiatives to improve their health in Kenya [28] and who should be a source of clarity for public doubts and misunderstandings about contraceptives appear to harbor and drive these same misconceptions. There is clearly a critical need for intensified evidence-based education about SRH and contraception for adolescents in these communities to dispel these misconceptions about contraception and sexual communication. Such interventions should also be cognizant of the value for child-bearing in certain communities and how this influences contraceptive use and acceptance.

A recurrent theme in our findings was the inability of parents to initiate and explicitly hold SRH discussions with their adolescents. This is a widely documented phenomenon that is commonplace in SSA where society generally discourages open discussions about SRH and its related topics, especially between adults and younger people [22, 29, 30]. Conversely, adolescents also reported their own fears and discomfort in speaking to parents about SRH and contraception. This is a problematic, albeit ubiquitous situation in SSA. Interestingly, parents (and other adults) only began to communicate explicitly about contraception after a pregnancy had occurred, much to the displeasure and detriment of their adolescents as this appears a belated intervention. In fact, it seemed counter-productive that parents felt that discussing SRH and contraception with adolescents was a covert encouragement to be sexually liberal but would discuss it with them after they were obviously engaged in risky sexual behaviour. At this stage, most sexual attitudes would have been formed and challenging to modify [31].

Indeed, an interesting paradox that arose in the study was that whilst on one hand, parents felt that discussing SRH and contraception with their wards would encourage sexual promiscuity, they would on the other hand prefer for their wards to get pregnant than to use (hormonal) contraception. This seems to suggest that fear of side effects and health concerns including concerns of infertility is ranked a more negative outcome in these regions than adolescent pregnancy and its attendant issues. As an important covariate of contraceptive use, desire for large family size remains a major driver to the ever-increasing populations in SSA and the attendant slow uptake of contraceptives in the region. Fertility remains a highly prized possession with driving factors such as religious beliefs and social structures that accord both spiritual and socio-economic rewards to high fertility [32, 33]. Some demographers have largely attributed the challenges of contraceptive programs including low uptake, widespread misconceptions and discontinuation to these pervasive ideals such as the widespread desire for large families [34, 35].

This study reflects the previously reported trends as the most widely reported concerns of respondents centered around the perceived negative side effects of contraception (especially hormonal) specifically related to the sterility of adolescents and congenital deformities of neonates. Evidently, more focus needs to be placed on interventions that clearly disabuse the perception of communities about contraceptives and their attendant side-effects especially with respect to fertility. There are gaps in the knowledge of community health volunteers and adolescents alike on the different modes of contraception, their duration of use and their modes of action. This is evidently a challenge that critically needs to be addressed. As responses from the participants suggested a general mistrust of “family planning drugs”, it appears that these communities may be better served with barrier methods. This actually provides an additional benefit of protection from STIs transmission, which was an additional concern expressed by some parents.

The presence of responses from some adults who held divergent views about contraception suggests a paradigm shift in adults’ stance about adolescents SRH. As, times change adults are recognizing their interactive (as opposed to autocratic) role in adolescents’ SRH and its importance for health outcomes. This increasing positive outlook of parental-adolescent communication about SRH has been reported in a few studies. In the study by Kibombo and colleagues, the authors found that whilst some adults found it difficult and did not deem it necessary to communicate with adolescents about SRH, some adolescents regarded the communication of adolescents especially with knowledgeable adults, essential for positive adolescent health [36]. Another study among different races in the United States found that the satisfaction of adolescents with their maternal relationship, and their assessment of parental approval with engagement in sexual intercourse was a key predictor for sexual behavior and contraceptive use [37]. These findings provide evidence of a ripe opportunity for CHVs to target parents with well-timed and helpful adolescent SRH information and services whilst encouraging interactions and conversations with their adolescents about sex from an early age. It is also important to note that most of the adults who held divergent views had undergone value clarification training or been exposed to SRHR programs that acknowledged adolescent sexuality and took a more positive and accepting approach to adolescents use of contraceptives. This demonstrates the importance of value clarification, training and exposure in changing adults’ perceptions towards adolescent sexuality and contraceptive use.

### Study limitations

This study was not without limitations, one being that our focus on adolescent girls may have neglected the views of adolescent boys on contraceptive use. Also, our sample only included adolescents aged 15–19 years and hence, did not capture the views of adolescent girls aged below 15 years who might have been sexually active. Another limitation is that responses might have been influenced by social desirability bias. However, respondents in the study came from dissimilar regions with significant geographical non-proximities and great diversities in terms of culture and ethnicity. Additionally, the consistency of our findings with other studies on contraceptive narratives among adolescent girls and adults support this study’s validity.

## Conclusion

Intensified well-informed family and community discussions on contraception are essential in providing accurate information on how contraceptive methods work, the side effects of each method and the benefits associated with them. Information on the differences between facts and myths about contraception should be targeted at CHVs to effectively prepare them in addressing pertinent concerns devoid of personal perceptions and bias. Media efforts should also be intensified to present socially-acceptable yet scientifically sound SRH information, especially on contraceptive use and side effects in relation to fertility. Additionally, programmatic efforts should be steered towards the provision of a balanced contraceptive mix of hormonal and barrier methods to encourage young users to select their more desired or suited options. The slightly higher acceptance of condom use compared to hormonal contraceptives may also provide an entry point to increase acceptance as well as further the conversation on supporting adolescents to experience sexuality more safely.

## Data Availability

The datasets used and/or analyzed in this study are available from the corresponding author on reasonable request.

## Abbreviations

CHV: Community health volunteer
CPR: Contraceptive prevalence rate
DREAMS: Determined, resilient, empowered, AIDS-free, mentored and safe
FGD: Focus group discussion
IDI: In-depth interview
ITH: In their hands
KDHS: Kenya demographic and health survey
SRH: Sexual and reproductive health
STIs: Sexually transmitted infections
SSA: Sub-Saharan Africa
